# Galvanic Forceps

**Published:** 1843-04

**Authors:** 


*Galvanic Forceps.*—These forceps are made by Gorck, the instrument maker, by order of Dr. Kilian, only to see what might be their effect upon the uterus. The blades are made of copper and zinc, and the metals are properly isolated from the hand of the accoucher. The first experiment with the galvanic forceps was made upon a woman aged 27, of dry constitution, choleric temperament, and jaundiced complexion. The application of the forceps was decidedly indicated in this case. The head of the child, which was in the first position, remained fixed at the lower aperture of the pelvis; and the torpidity of the uterus was so great, that the child had not moved for two hours and a half; while the infiltration of the scalp was of the size of a man's fist. Before applying the forceps, Dr. Kilian had the patient bled to fourteen ounces; but this had no influence on the action of the uterus. The blades were easily introduced into the uterus; but the moment they were joined, the woman had a fresh pains which was very violent, without being unbearable. At the same time a movement was felt in the whole uterus, which became as hard as a stone, and lost the morbid sensibility which it had shown before on each examination.

This state of things continued from the beginning to the end of the application of the forceps, and in spite of the hardness of the uterus the pains had no expulsive power. Nothing, however, indicated any spasm of the internal sexual system. After four actions with the forceps, the head cleared the lower aperture of the pelvis, and then (as well as before), the femoral muscles underwent a spasm and trembling of an unprecedented kind. Dr. Kilian then removed his hands from the instrument, to see if the uterus, which was still contracted, would not complete the expulsion of the child's head; but this was not the case, so that he was obliged to continue the use of the forceps.

The infant immediately breathed, which was surprising, when we consider how long it had been fixed in the lower aperture of the pelvis. Hardly were the shoulders free, when the child, which was very strong, began to cry, and the pulsation of the cord immediately ceased. The uterus then contracted, and in five minutes the placenta was in the vagina. There were no pains after delivery, and the lying-in was quite regular.

*N. Y. Lancet* from *Gazette des Hôpitaux.*

